# Advanced Nanomedicine for High-Risk HPV-Driven Head and Neck Cancer

**DOI:** 10.3390/v14122824

**Published:** 2022-12-19

**Authors:** Qiang Xu, Ye Chen, Yuan Jin, Zhiyu Wang, Haoru Dong, Andreas M. Kaufmann, Andreas E. Albers, Xu Qian

**Affiliations:** 1 The Cancer Hospital of the University of Chinese Academy of Sciences (Zhejiang Cancer Hospital), Institute of Basic Medicine and Cancer (IBMC), Chinese Academy of Sciences, Hangzhou 310022, China; 2 Department of Clinical Laboratory, The Cancer Hospital of the University of Chinese Academy of Sciences (Zhejiang Cancer Hospital), Institute of Basic Medicine and Cancer (IBMC), Chinese Academy of Sciences, No. 1 East Banshan Road, Gongshu District, Hangzhou 310022, China; 3 Wenzhou Medical University, Wenzhou 325000, China; 4 Clinic for Gynecology, Berlin Institute of Health, Charité-Universitätsmedizin Berlin, Freie Universität Berlin, Humboldt-Universität zu Berlin, 12203 Berlin, Germany; 5 Department of Clinical Medicine, Oto-Rhino-Laryngology, Medical School Berlin, 14197 Berlin, Germany

**Keywords:** HPV-related cancer, oropharyngeal cancer, cancer vaccine, nanotechnology, immunotherapy, photoimmunotherapy, cancer nanomedicine

## Abstract

The incidence of high-risk Human Papillomavirus (HR-HPV)-driven head and neck squamous cell carcinoma (HNSCC) is on the rise globally. HR-HPV-driven HNSCC displays molecular and clinical characteristics distinct from HPV-uninvolved cases. Therapeutic strategies for HR-HPV-driven HNSCC are under investigation. HR-HPVs encode the oncogenes E6 and E7, which are essential in tumorigenesis. Meanwhile, involvement of E6 and E7 provides attractive targets for developing new therapeutic regimen. Here we will review some of the recent advancements observed in preclinical studies and clinical trials on HR-HPV-driven HNSCC, focusing on nanotechnology related methods. Materials science innovation leads to great improvement for cancer therapeutics including HNSCC. This article discusses HPV-E6 or -E7- based vaccines, based on plasmid, messenger RNA or peptide, at their current stage of development and testing as well as how nanoparticles can be designed to target and access cancer cells and activate certain immunology pathways besides serving as a delivery vehicle. Nanotechnology was also used for chemotherapy and photothermal treatment. Short interference RNA targeting E6/E7 showed some potential in animal models. Gene editing by CRISPR-CAS9 combined with other treatments has also been assessed. These advancements have the potential to improve the outcome in HR-HPV-driven HNSCC, however breakthroughs are still to be awaited with nanomedicine playing an important role.

## 1. Introduction

High-risk human papillomavirus (HR-HPV)-related head and neck squamous cell carcinoma (HNSCC) is found in all anatomical sites of the head and neck, especially in the oropharyngeal region (OPSCC) [1]. Incidences of HR-HPV-driven OPSCC are rising, while those of tobacco use and alcohol consumption are decreasing worldwide [1]. Compared with HPV-negative OPSCC, HR-HPV-driven OPSCC exhibits different genomic features, nonkeratinized histology, an increased and early tendency toward lymph node metastasis, higher sensitivity to conventional treatment and, as a result, a better clinical prognosis, as reviewed elsewhere [1,2]. The 8th edition of the American Joint Committee on Cancer (AJCC) revised the tumor node metastasis classification (TNM) staging system of HPV-associated OPSCC and suggested a de-intensified treatment for these patients if the neck staging is correct [3]. Recommendations on HPV-related OPSCC treatments and achievements from ongoing research have been updated in recent National Comprehensive Cancer Network (NCCN) Guidelines [4]. However, many challenges remain in the development of a de-escalation protocol of conventional treatment. For example, randomized phase III trials have demonstrated that cetuximab plus radiation therapy (RT) does not exhibit superior effects compared with cisplatin plus RT [5]. One reasonable explanation is that these treatments were generally not well tolerated. Although dose-reduced RT may minimize functional impairment of the salivary glands, the swallowing tract and neck tissues with a similar progression-free survival in p16-positive OPSCC, there is a lack of randomized phase III trials to date to validate these findings [4]. Additionally, HPV testing by p16 immunohistochemistry (IHC) alone as the biomarker determinizing HPV-related HNSCC has some limitations. As analyzed in a previous meta-analysis, p16+/HPV-HNSCC may characterize a new relevant HPV-independent subtype [5]. Immune checkpoint inhibitor (ICI) trials have demonstrated inferior effects in both p16+ and p16- HNSCC in different trials (KEYNOTE-012, KEYNOTE-048, and KEYNOTE-040). A recent study found high CD103+ intratumoral immune cell expression was evaluated to stratify patients with low-risk (the low-risk subgroup was defined by Ang et al. in RTOG 0129 clinical trial regarding to the risk of death) HPV-associated OPSCC who received cetuximab plus RT for superior prognosis [6,7]. While new, possibly more reliable biomarkers are under evaluation for selecting patient cohorts who will benefit from de-escalation treatment, improving the current treatment protocol and developing new therapeutic regimens, such as targeting HPV E6/E7 or vaccines, which are equally important. 

Advances in material science, nanotechnology, and CRISPR technology can now provide platforms to study combinations of current conventional therapy with new therapeutics aimed at specific biological targets on cancer cells [8,9]. These approaches demonstrate the advantages of site-specific drug delivery with reduced toxic effects that can widen the therapeutic window or improve the therapeutic index [9,10,11,12,13,14]. In this review, we illustrate current technological innovations for HPV-related HNSCC treatments and discuss promising new insights for new therapeutic targets.

## 2. Cancer Biology

In this review, we define HR-HPV-driven HNSCC as HPV-induced carcinogenesis mainly driven by two HPV oncogenes (E6 and E7) while HPV-related HNSCC may include the cases detected as an innocent bystander of concurrent HPV infection [1,5]. The carcinogenic activities of E6 and E7 proteins are related to the increased degradation of p53 and Rb, respectively, resulting in genomic instability and resistance to apoptosis [1]. The Cancer Genome Atlas (TCGA) analysis shows that *TP53* mutations and *CDKN2A* gene changes are common in HPV-negative HNSCC but are rare in HR-HPV-driven tumors [15]. Genome changes in HR-HPV-driven tumors mainly include mutation of the helical domain of the *PIK3CA* oncogene, frequent deletion of *TRAF3*, and amplification of the cell cycle-related gene *E2F1* (TCGA) [15]. In addition, higher viral apolipoprotein B mRNA editing catalytic polypeptide-like (*APOBEC*) mutation rates were found in HR-HPV-related OPSCC with a high *APOBEC* mutation burden by analyzing paired human somatic and HPV genomes, which suggests that *APOBEC* mutagenesis is involved in HR-HPV-driven carcinogenesis [16]. The off-target *APOBEC* activity in HR-HPV-driven OPSCC may lead to driver mutations such as *PIK3CA* [17,18]. These genetic and epigenetic alterations induced by HR-HPV infection are believed to lead to HR-HPV-driven cancer. However, the underlying mechanism remains elusive.

In HR-HPV-driven cancer, HPV-specific antitumor host immune responses are also observed, as reviewed in many publications [2,19]. These findings have stimulated much optimism and research into stimulating and modifying the natural host immune response to control the growth and spread of cancer cells or to even kill them. However, HPV-driven cancer is able or evolves to evade by several mechanisms of immune surveillance that allow the malignancy to become established in the host and to form metastases. Known immune evasion mechanisms are downregulation of major histocompatibility complex (MHC)-I expression, passive immune evasion by suppressing antigen presentation during both the early and late phases of infection in epithelial layers, and E6/E7 oncoprotein-mediated blockade of immune-related genes or signaling pathways [19,20]. Within the tumor microenvironment (TME), tumor infiltrating lymphocytes (TILs) have been shown to be associated with the survival of many different tumor types, including HNSCC. In most HR-HPV-related OPSCCs, there are usually more TILs and a better prognosis than in HPV-negative tumors. However, the survival rate of low-level TILs in HR-HPV-related patients was low, similar to that of HPV-negative HNSCC patients. In addition, the decrease in TIL permeability affected by tobacco exposure is associated with late and local recurrence [21,22]. In an analysis of the transcriptional signatures of immune cells in the TME, HR-HPV-related HNSCC demonstrated different helper CD4+ T-cell and B-cell transcriptional signatures compared with HPV-negative tumors [23]. PD L1 expression was found to be more frequent in HR-HPV-related OPSCC and associated with TILs compared with HPV-negative tumors [18,24]. Interestingly, one study revealed HR-HPV genome integration in the immune checkpoint genes PD L1 or PD L2, which led to elevated expression of such genes in 3 of 73 HR-HPV HNSCC samples [24]. In HPV-related HNSCC, virus-specific CD8+ T cells directed against epitopes from HPV E2, E5, and E6 expressed PD1 and were found in CD8 TILs in the TME. Among those TILs, three subsets of PD1+ stem cells and transitory and terminally differentiated TILs were found [25]. HPV-specific PD1+ stem-like CD8+ T cells are more frequent in the TME but very rare in the circulation [25]. HPV-specific B-cell responses have also been reported in HPV-related HNSCC as localized HPV-specific IgG antibody-secreting cells against E2, E6, and E7 HPV proteins in the TME [26]. In addition, analyzing the data from The Cancer Genome Atlas (TCGA) portal, HPV-related non-OPSCC tumors in other anatomic subsites of the head and neck region demonstrated different immunogenomic features, i.e., lower estimated scores of B cells and CD8+ T cells and higher estimated scores of cancer-associated fibroblasts and M2 macrophages compared with HPV-related OPSCC [27]. These findings add to current knowledge of the biology of HPV-related HNSCC and help to develop potential therapeutic options regarding immunotherapeutic approaches such as HPV therapeutic vaccination.

Cancer stem cells (CSCs), a small group of stem-like cells in tumors, play a critical role in HNSCC development, recurrence, and metastasis [28]. An early study reported that HPV-related HNSCC had a higher proportion of CSCs than HPV-negative HNSCC, which may be attributed to p53 inactivation by HR-HPV [29]. CSCs are the cell population that is most resistant to radiation and chemotherapy. Despite the fact that in HR-HPV HNSCC, CSCs are found at a high proportion in primary tumors and their metastases, which occur earlier than in non-HPV-related HNSCC, this is no contradiction to the clinical observation that HR-HNSCC and their CSCs are generally more susceptible to therapy. HR-HPV HNSCC maintains an intact apoptotic pathway through p53 that is successfully activated by radiation. This may explain why HPV-related HNSCC, which does not have higher ALDH expression than HPV-negative HNSCC [30], may still be more therapy resistant, as clinically observed due to mutation-inactivated p53. Deregulation of p53 by the HR-HPV E6 protein may reverse NOTCH and NANOG inhibition, leading to stemness features [31]. In a cervical cancer model, overexpression of the HPV oncoprotein E6 can maintain the stem-cell phenotype and stemness of CSCs through upregulation of Hes1, and short interfering RNA (siRNA) silencing of E6 or Hes1 leads to redifferentiation loss of self-renewability/stemness of CSCs [32]. Moreover, the HPV receptor integrin alpha 6 (ITGα6) regulates the stemness phenotypes of HR-HPV HNSCC cells partially by mediating the AKT pathway [33]. For more detailed discussions, please refer to recent reviews [31,34]. Using CD44+/ALDH+ as CSC markers, CSC proportions in both HPV-related and HPV-negative cell lines were elevated after irradiation [35]. While further studies on various CSC markers with expanded sample sizes are necessary, it is important to elucidate the immunity of CSCs in HPV-driven HNSCC that can help to develop novel therapeutic strategies.

Alternative HPV splicing has been reported to be involved in the carcinogenesis of cervical cancer and HNSCC; for example, RNA-binding proteins that control HPV RNA processing can be provided as potential targets (see a recent review) [36]. A recent study revealed that the ecdysoneless (ECD) protein could interact with the HPV E6 protein and regulate E6/E7 RNA splicing to promote HR-HPV-driven oncogenesis [37]. In summary, HPV-related HNSCC shares specific biological causes and characteristics that provide targets on which research for future therapies currently focuses.

## 3. Vaccine

Therapeutic vaccines for HR-HPV-driven cancer have been investigated in many preclinical studies and clinical trials [38]. While most of these early efforts targeted cervical cancer, HR-HPV HNSCC is attracting increasing attention as its incidence continues to rise globally. Vaccines with tumor-specific or tumor-associated antigens aim to stimulate an immune response and induce cytotoxic activity against tumor cells. Several different types of viral and nonviral antigens have been explored for this effect. In particular, for HR-HPV HNSCC, the viral oncoproteins E6 and E7 offer promising antigen targets, although there was a recent report suggesting that HPV E2 and E5 should also be considered for inclusion [25].

**Figure 1:**
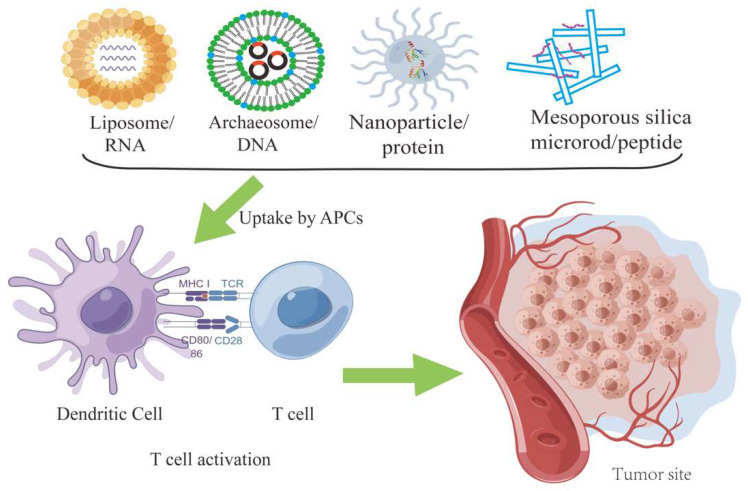
Various types of nano carriers have been constructed for the delivery of different cargos to antigen presenting cell to activate T cells. Several nano vaccines are shown here. The figure is drawn by Fig-draw.

Various types of strategies utilizing DNA [39,40], RNA [41,42], protein [43,44], peptide [45,46,47,48] or tumor or cell lysate [49], and tumor-derived autophagosomes [50] have been explored with various degrees of success (Figure 1). While several clinical trials for cancer of the cervix uteri have completed phase III, all the therapeutic vaccines for HNSCC in clinical trials are currently at the stage of clinical phase I or II trials. Supplemental Table S1 provides an overview of ongoing clinical trials. For example, MEDI0457 (also named INO-3112) [51], a DNA vaccine with a synthetic plasmid targeting E6 and E7 of HPV-16/18 and IL-12, was tested in combination with the PD-L1 targeting antibody durvalumab in a phase Ib/II clinical trial for patients with recurrent/metastatic HPV-associated HNSCC (NCT03162224). Another vaccine in trial ADSX 11-101 uses a live attenuated, recombinant Listeria monocytogenes bacterium that has been bioengineered to secrete an antigen-adjuvant fusion protein that includes a truncated fragment of listeriolysin O fused to the full-length E7 peptide of HPV-16. A phase II clinical trial (NCT02002182) on HR-HPV OSCC is expected to be completed in 2023.

By itself, the effect of these vaccines against advanced HNSCC may be limited due to the same suppressive tumor microenvironment that immune checkpoint blockers face. The transport and targeting of antigen, adjuvant, and other therapeutic agents also needs to be optimized to achieve synergistic effects. Using properly constructed nanocarrier systems as delivery vehicles may offer a suitable path to overcome these challenges [52,53].

Nanocarriers are designed to protect their cargo from destruction and to deliver it to a target-defined destination. In this manner, the bioavailability of the drugs is high at the intended target and may remain low elsewhere. Significant progress has been made in recent years, and two nanocarrier-based vaccines have entered clinical trials. Table 1 and Table 2 summarize the preclinical and clinical studies on nanocarrier-based vaccines.

Kranz et al. demonstrated that cationic lipid carriers can be formulated to protect RNA from degradation and improve the uptake efficiency by dendritic cells and macrophages [54]. Administration of such a liposomal HPV16 mRNA formulation (RNA-LPX) elicits a robust E7 antigen-specific CD8+ T-cell response in HPV-positive TC-1 and C3 tumor murine models [41]. Analysis of tumor samples from vaccinated mice revealed upregulation of proinflammatory molecules, DC activation markers, markers for monocyte/macrophage recruitment, and immune checkpoint molecules, indicating that vaccination was associated with polarization toward a proinflammatory and less immune-suppressive contexture. The combination with anti-PD-L1 treatment resulted in complete remission of the tumors in 10 of the 15 TC-1 tumor-bearing mice and improved overall survival to approximately 70% from less than 10% for anti-PD-L1 alone and 30% for the RNA-LPX vaccine alone. The RNA-LPX-based vaccine BNT113 is currently in a phase II clinical trial (NCT04534205 & NCT03418480) for HR-HPV HNSCC. The synergy between the E7 RNA-LPX vaccine and local radiotherapy was also investigated recently in a preclinical model [55]. It was demonstrated that the combination of E7 RNA-LPX and local radiotherapy showed potent therapeutic effects exceeding those of either monotherapy.

A nanocarrier can also be designed to trigger various immune pathways to enhance immunological effects. The lipids that form the nanoparticles can also serve as potent adjuvants. It was reported that the cationic lipid enantiomer R-1,2-dioleoyl-3-trimethyl-ammonium-propane (R-DOTAP) stimulates endosomal TLRs, resulting in Myd88-dependent production of type I IFN [56]. R-DOTAP nanoparticles containing HPV-16 E7 peptides resulted in a potent antigen-specific CD8 T-cell response and antitumor activity in a mouse model with implanted TC-1 tumors. The R-DOTAP-based HPV vaccine PDS0101 in combination with ICB is now in a phase I/II clinical trial (NCT05329532, NCT04287868) [47]. Miao et al. conducted a combinatorial library search of ionizable lipids for their mRNA delivery vehicle and found that the top-performing lipids induce APC maturation via the intracellular stimulator of interferon genes (STING) pathway and demonstrated enhanced antitumor efficacy [42].

Nanocarriers can also be engineered to codeliver proper adjuvants to induce synergistic immune activation. For example, Kuan et al. loaded two Toll-like receptor agonists, MPLA and CpG, onto synthetic high-density lipoprotein (sHDL) nanodiscs (NDs) [57]. This adjuvant system combined with the HPV-16 E7 peptide (E7+ND-MPLA/CpG) elicited stronger E7-specific CD8+ T-cell responses in tumor-bearing mice than soluble E7+MPLA/CpG. By this treatment, complete regression of established TC-1 tumors in all treated animals was achieved. This sHDL ND system was also used in a vaccination trial against aldehyde dehydrogenase (ALDH), which is a marker for cancer stem cells [58]. Vaccination against ALDH combined with anti-PD-L1 therapy prolonged overall survival in murine models of melanoma and breast cancer. Given the high expression of ALDH in HNSCC stem cells [59], this new immunotherapy approach may also hold promise for the treatment of HNSCC.

Mesoporous silica microrods (MSRs) are another interesting platform for delivering vaccines and adjuvants [60,61]. MSR carries antigen and adjuvant by absorption and thus does not require modification of the antigen. Li et al. showed that injection of a vaccine composed of MSR absorbed with polyethyleneimine (PEI) and H16-E7 peptides can eradicate large established tumors in ~80% of mice with TC-1 tumors and generate immunological memory [60]. Manganese (Mn4+)-doped silica nanoparticles were also explored as vehicles for peptide-base vaccines, where the nanoparticles served as self-adjuvants [62].

Other types of nanocarriers include nanosatellites formed by polymer-coated iron oxide cores with inert gold satellites [63], self-assembled peptide-formed nanofibers or nanoparticles [64,65], virus-like particles [66], and bacterial outer membrane vesicles [43]. Magnetic nanoparticles that include iron oxide can also be used for tracking dendritic cell migration using magnetic resonance imaging [67]. It was shown that MRI intensity in lymph nodes correlated with DC trafficking and can identify vaccine responders early.

In another interesting work, photosensitizer-induced HPV16 E7 nanovaccines were constructed by linking bovine serum albumin with the E7 antigen and then encapsulating the photosensitizer and adjuvant through disulfide bonds [68]. Infrared laser irradiation of photosensitizers produced a photo-oxygen response that induced the maturation of dendritic cells and stimulated T-cell effects. The enhanced antitumor effect was demonstrated in TC-1 tumor-bearing mice.

The effect of the vaccine on tumor recurrence after surgery for HNSCC was also investigated in a recent report [69]. In this study, mEERL95 cells were injected into the submental space of mice to generate a tumor model. The HPV E7 peptide vaccine only had a marginal effect on tumor growth or overall survival for mEERL95 tumors, as vaccine-induced CD8+ T cells can only poorly infiltrate this type of tumor. However, the vaccine injected prior to surgery successfully prevented tumor recurrence in all the mice, while unvaccinated mice had a 60% recurrence rate. All vaccinated mice survived until the end of the experiment, but only 35% of the unvaccinated mice survived.

In addition to antigens such as HPV-E6, E7 or NANOG [70] that are shared by many patients, therapeutic vaccines can also be engineered to target patient-specific antigens [71,72]. However, preparing such personalized vaccines is labor, time, and cost intensive, and the effects are difficult to analyze scientifically. Thus, an alternative approach called in situ vaccination has been developed, which has the advantage of being both personalized and off-the-shelf [73]. In this approach, the death of tumor cells caused by methods such as radiation, chemotherapy agents or photothermal therapy leads to the release of tumor-associated antigens, which are then processed and presented by APCs. Immunomodulators can be administered at the same time to enhance particular steps of this process. Several phase I or phase II clinical trials (NCT02643303, NCT02423863, NCT03789097) of in situ vaccination involving HNSCC have been carried out. Many carefully designed nanocarriers have been utilized for the delivery of immunomodulators for in situ vaccines. Chen et al. constructed lipidoid nanoparticles that can promote cross-presentation of tumor-associated antigens (TAAs) and deliver 2′5′-3′5′ cyclic guanosine monophosphate-adenosine monophosphate (cGAMP) to activate the STING pathway, resulting in an enhanced antitumor effect [74]. Wang et al. injected a genetically attenuated strain of Salmonella coated with antigen-adsorbing cationic polymer nanoparticles to promote the accumulation of antigen at the tumor periphery [75]. Hollow mesoporous organosilica nanoparticles were used to transport Annexin A5 in another study [76]. Release of Annexin A5 in both the oxidative TME and bioreductive intracellular environment blocks immunosuppressive apoptosis and promotes immunostimulatory secondary necrosis. While these nanocarrier-enabled in situ vaccines have not specifically targeted HNSCC, they represent an alternative path for therapeutic vaccine development.

## 4. Gene Silencing and Editing

The growth of neoplasms often depends on a certain set of genes, many of which were identified in previous studies. One viable approach to induce tumor regression in cancer patients is to suppress these genes through gene silencing and editing. In HPV+ head and neck cancer, the genes of choice are usually the viral oncogenes HPV E6 and E7. Gene silencing and editing are often carried out through RNA interference (RNAi) or CRISPR/Cas9.

One potential pitfall for such an approach targeting E6/E7 is whether HR-HPV HNSCC can become independent of HPV when HPV viral gene expression is suppressed. HPV E6/E7 oncogenes were not expressed in up to 50% of HPV DNA-positive HNSCC [77,78]. Such HPV-inactive HNSCC has a worse overall survival and higher recurrence rate than HPV-active HNSCC [79]. In a recent study, Abboodi et al. [80] reported that in HPV-16-transformed keratinocytes, knockout of p53 by CRISPR/Cas9 resulted in a 5-fold decrease in E7 mRNA levels and suggested that HPV-16-transformed cells can lose dependence on the continuous expression of viral oncogenes for proliferation. HPV-inactive HNSCC often has mutated p53, while it is rarely mutated in HPV-active HNSCC. Whether knockdown or knockout of HPV E6/E7 in HPV-dependent cells with wild-type p53 can give rise to cells that are HPV inactive but can still proliferate is unknown and needs to be carefully monitored.

### 4.1. RNA Interference

Sequence-specific RNA templates can be designed to guide the intended knockdown of the targeted genes in RNAi. While most of the RNAi studies against HPV are carried out in cervical cancer cells or animal models, several studies have demonstrated that in the HR-HPV HNSCC cell line, E6- and/or E7-targeting shRNA or siRNAs can downregulate E6 and E7 and upregulate the expression of p53 and pRb proteins [81,82,83]. A significant inhibitory effect of E6 and/or E7 siRNA compared with those of control groups was observed both in vitro and in vivo [82]. To fully realize the therapeutic potential of the RNAi method, improved delivery vehicles are needed. In a recent study, lipid nanoparticles coated with anti-epithermal growth factor receptor antibodies were used to deliver anti-E6/E7 siRNA [84]. This approach demonstrated significant suppression of viral oncogenes and induction of apoptosis, resulting in antitumor activity both in vitro and in vivo.

### 4.2. CRISPR/Cas9

While RNAi can knock down the targeted mRNA, the presence of the relevant gene in the genome is not affected, so constant interference is required for treatment. To permanently knockout the targeted genes, gene editing tools such as CRISPR/Cas, zinc finger nucleases, or transcription activator-like effector nucleases are needed. The CRISPR/Cas9 system has emerged as the most promising tool for genome editing due to its robustness and versatility. To fully reach the therapeutic potential of the CRISPR/Cas9 system, safe and efficient delivery vehicles must be developed, and liposomes or lipid nanoparticles may provide the best option [85]. The CRISPR/Cas9 system can also come in different forms, such as plasmid DNA, mRNA, or ribonucleoprotein, each with its own advantages and challenges. Various lipid compositions have been explored in numerous studies to obtain the desirable effects of prolonging circulation time, improving targeted delivery and cellular uptake, reducing immunogenicity, and minimizing toxicity. The interim results from the first clinical trial with lipid nanoparticles as delivery vectors for CRISPR/Cas9 demonstrated successful knockout of the targeted gene with only mild adverse effects for the treatment of hereditary transthyretin amyloidosis with polyneuropathy [86].

For HR-HPV HNSCC, the HPV E6/E7 genes are again logical choices as targets for genome editing to suppress tumor growth. Cas9 and sgRNAs specific for E6/E7 can cause inactivation of the E6/E7 genes in cervical cancer cells, resulting in induction of p53 or Rb and leading to cell cycle arrest and eventual cell death [87,88,89]. Similar approaches also resulted in an inhibition of tumor growth in nude mice inoculated with Cas9/sgRNA-treated cervical cancer cells [89]. It is worth noting that nearly all cervical cancers are HPV-dependent, so concern regarding the HPV inactive form is not as grave as in HNSCC cases. The synergistic effect of E6/E7 knockout and chemotherapy [90] or PD1 blockade [91] was also explored. A phase I clinical trial (NCT03057912) was also carried out to assess the safety and efficacy of CRISPR/Cas9 in the treatment of HPV-related cervical intraepithelial neoplasia I. In HR-HPV OPSCC, it was reported that targeting E7 alone did not affect cell viability, while targeting E6 and E7 simultaneously resulted in a 50% loss of cell viability [92]. In these OPSCC cells, CRISPR/Cas9-mediated loss of E7 restored the cGAS-STING response, so it was suggested that the combination of E7 knockout may be combined with a STING agonist to induce favorable antitumor effects.

In addition to virus-based vectors [93,94], lipid nanoparticles can also be used to deliver CRISPR/Cas for editing HPV genes. Jubair et al. used PEGylated liposomes as vehicles for the in vivo delivery of CRISPR/Cas9 targeting E7 in a cervical cancer xenograft mouse model, resulting in tumor elimination and a significant survival advantage [95,96]. Poly (β-amino ester)-based polyplex nanoparticles were utilized in other studies to deliver CRISPR/Cas9 recombinant plasmids targeting the HPV16 E7 oncogene, which inhibited xenograft growth in a mouse model when administered via peritumoral injection [97,98,99].

Nanoparticles can be designed to deliver the cargo more specifically to the tumor site. In a proof-of-concept study, Tang et al. designed phenylboronic acid (PBA)-derived lipid nanoparticles to deliver Cas9 mRNA and HPV E6-targeting sgRNA preferentially to cancer cells through the interaction between PBA and sialic acid on the cell surface [100]. An 18.7% insertion-deletion of the HPV18E6 gene and a 50% decrease in cell viability were observed in treated HeLa cells. In another study, pH-responsive cationic liposomes with strong tumor targeting and gene knockout efficiency were designed to deliver CRISPR/Cas9 [101]. Intratumoral injection of such cationic liposomes effectively inhibited the proliferation of HPV16+ cervical cancer cells and induced apoptosis by inactivating the E6/E7 oncogene in nude mice. In a different approach, Lao et al. proposed that a carrier with a lower charge density may be a better choice for Cas9 plasmid delivery, as they hypothesized that a high charge density carrier may lead to sustained Cas9 expression and result in more off-target effects [102]. They designed a self-assembled micelle composed of quaternary ammonium-terminated poly (propylene oxide) (PPO-NMe3) and amphiphilic Pluronic F127 optimized for delivering the Cas9 plasmid to knock out HPV16 E7, which led to significant inhibition of HPV-induced cancerous activity both in vitro and in vivo.

While the CRISPR/Cas9 system has shown tremendous potential in the treatment of HR-HPV-related cancers, it is still limited by a low efficiency of editing and nonnegligible off-target effects. To overcome these difficulties, a modified Cas9 system [103] or other related enzymes, such as Cas13 [104], was also explored and demonstrated to significantly improve the gene knockout efficiency or specificity of HPV oncogenes. How well such systems work in vivo and which delivery vehicles will be most suitable remain to be investigated.

### 4.3. p53

The HPV oncogene E6 drives the carcinogenic process mainly by promoting the degradation of p53. Therefore, upregulating p53 expression or restoring p53 bioavailability by gene editing may have potential as a therapy for HR-HPV-driven cancers [105]. Early attempts to reactivate p53 function focused on using adenoviral vectors to deliver wild-type p53. Such efforts resulted in Gendicine (approved by state FDA in China) [106], the first gene therapy product with a combination of human wt *p53* gene and adenovirus serotype-5 vector (Ad5) in 2003 for the treatment of head and neck cancer, and—later on—other types of cancer. In an early clinical trial, 42 patients with nasopharyngeal carcinoma were treated with Gendicine combined with standard radiotherapy compared to the control group of 40 patients received only radiotherapy [107]. While the group receiving Gendicine demonstrated higher complete response rate (66.7% vs. 24.4%, *p* = 0.01) and lower 5-year recurrence rate (2.7% vs. 28%, *p* = 0.002), the benefit for 5-year overall survival rate and 5-year disease free survival rate are not statistically significant. The benefit for long-term overall survival rate remains to be established as seen in clinical trials summarized by Zhang et al. [106]. The development of the CRISPR/Cas9 system has provided new tools for accurate and safe gene manipulation. It was proposed that CRISPRa/dCas9, a variant of the CRISPR system, can be utilized for activation of p53 [105].

## 5. Extracellular Vesicles

Extracellular vesicles (EVs) are nanoparticles naturally released by cells that are associated with multiple pathological conditions, including cancer [108,109]. Biomolecules such as E6 oncoproteins, E6/E7 mRNA, and other molecules were identified in EVs of HR-HPV-driven cancer, including cervical cancer and OPSCC [110]. For example, fewer cancer-associated fibroblasts (CAFs) were infiltrated in HR-HPV HNSCC. This may be because the enriched miR-9 in EVs secreted from HR-HPV HNSCC cells could transfer into fibroblasts via EVs that can significantly reduce the phenotypic transformation of fibroblasts [111]. Moreover, HR_HPV HNSCC-derived exosomal miR-9 induces macrophage M1 polarization and increases tumor radiosensitivity [112].

**Figure 2:**
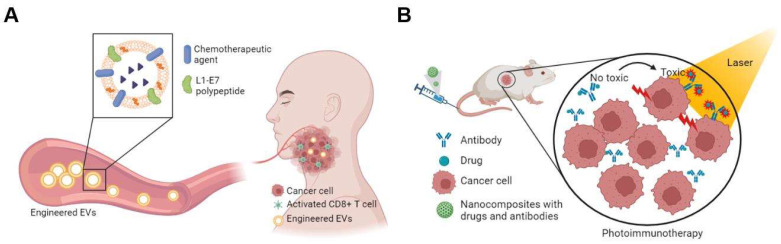
Application of extrasellar vesicles (EVs) in cancer vaccine (**A**) and nanocomposites in photoimmunotherapy (**B**).

Given the complex interaction of nanoparticles with the biological TME, which may lead to a protein corona and, therefore, failures, as seen in some clinical trials, engineering cell-released biological nanoparticles, EVs, as drug delivery systems is employed by taking advantage of their biofeatures [113,114]. Endogenously engineered EVs loaded with tumor antigen were developed as a therapeutic HPV vaccine [115]. In one study, the Nefmut/anti-HPV16-E7 scFv chimeric product was efficiently loaded in EVs, bound HPV16-E7, and inhibited the proliferation of HPV16-E7-expressing cells [116]. In another study, synergistic effects of exosomal crocin or curcumin compounds and the HPV L1-E7 polypeptide vaccine construct on tumor eradication were observed in a C57BL/6 mouse model [117]. With emerging EV biology, therapeutic EVs warrant further study (Figure 2A).

## 6. Targeting PI3K/AKT Pathway

As mentioned previously in Section 2, *PIK3CA* are frequent altered in HNSCC. Overexpression of PI3K often leads to hyperactivation of the PI3K/AKT pathway, which is vital for tumor development. Therefore, targeting PI3K and its substrates is considered a promising strategy for cancer treatment. Several small molecule PI3Kα inhibitors are now under investigation in clinical trials [118].

Small molecules inhibiting PI3Kα can also be packaged into nanocarriers to improve its efficacy and safety. To achieve targeted delivery of drug to the tumor site, Mizrachi et al. encapsulating BYL719, a PI3Kα inhibitor currently in clinical development, into dextran sulfate-based nanoparticles that targets P-selectin, a cell adhesion molecule often over-expressed in the vasculature of several human cancers [119]. In animal HNSCC models being established either by cell-line-based tumor or patient-derived xenograft, nano-particles carrying BYL719 demonstrated tumor growth inhibition and radio-sensitization effects which are similar to the effect of a seven-fold higher dose of BYL719 alone.

The combination of irreversible electroporation (IRE) with liposome-encapsulated NVP-BEZ235, a dual PI3K/mTOR inhibitor was also explored for HNSCC treatment [120]. For head and neck cancer xenografts in nude mice, this combination effectively eradicated the tumor masses, with no palpable or extractable tumor mass observed after two months, while either IRE alone or IRE + oral NVP-BEZ235 fail to achieve the same effects.

In another study, a small molecule inhibitor PHT-427 that targets AKT/PDK1, downstream substrates of PI3K, was loaded onto α-tocopheryl succinate-based polymeric nanoparticle and tested in hypopharynx squamous cells carcinoma [121]. Nanoparticles loaded with PHT-427 effectively suppressed AKT/PDK1 expression and produced high oxidative stress levels leading to apoptosis.

## 7. Combination Therapies

For HNSCC, the standard treatment option is surgery and radiation therapy for early-stage cancer, while surgery followed by a combination of systemic chemotherapy and local-regional radiation therapy for advanced disease is established. Given the distinct onco-genomics of HR-HPV-driven HNSCC compared with HPV-negative tumors, HR-HPV-dependent selective therapy warrants innovative approaches. Another important strategy is utilizing combination therapy to target cancer cells, especially with less harmful effects on healthy cells. Recently, a screening of 864 diverse compounds potentially causing cell death was performed on HNSCC cell lines and validated in patient-derived xenograft (PDX) models of HR-HPV-related OPSCC [122]. The authors found that aurora kinase inhibitors had a more profound effect on HR-HPV HNSCC that was Rb level-dependent. Moreover, in relation to Rb degradation caused by the oncoprotein E7, the mitotic checkpoint genes *MAD2L1* and *BUB1B* were highly expressed in HR-HPV HNSCC. Depletion of the *MAD2L1* regulator *TRIP13* can enhance Aurora kinase inhibition-induced apoptosis. Combining Aurora kinase inhibition with *TRIP13* depletion had synergistic effects on HR-HPV HNSCC but not HPV-negative HNSCC with intact Rb expression [123]. In addition, anti-E6/E7 siRNA coupled with anti-epidermal growth factor receptor (EGFR) antibodies delivered via targeted lipid-based nanoparticles (tLNPs) have shown successful antitumor effects in a xenograft HR-HPV HNSCC model [84]. These findings add to current HPV-specific treatment strategies and warrant further translational research. Additionally, as we have introduced in the above section, cancer vaccines such as the HPV16 E6/E7 RNA-LPX vaccine combined with pembrolizumab are currently being evaluated in a phase II clinical trial for patients with HPV16+ and PDL1+ HNSCC (NCT04534205).

Radiation is utilized to treat more than half of HNSCC patients; as a result, boosting the therapeutic ratio or improving the radiation impact in tumors while protecting the surrounding healthy tissues is a critical therapeutic goal [123]. The latest developments in nanomedicine make it possible to improve the efficacy of radiotherapy through radio-sensitization or radiation protection technology. For example, PEG-coated Au–Ag alloy nanoparticles (BNPs) are one example that can readily enter the cytoplasm of KB cells (derived from an epidermal carcinoma of the mouth) and have shown significant in vitro radio-sensitization with an enhancement ratio of 1.5–1.7 [124]. Standard radiotherapy enhancement for HNSCC is concurrent platinum-based chemotherapy. Prateek Bhardwaj et al. [60] successfully created a dual nanocarrier-in-hydrogel platform (PTX-CDDP-PH) to enhance the site-specific delivery of radiosensitizers such as cisplatin and paclitaxel [125]. Interestingly, the nanoparticles delayed the two chemotherapeutic medicines’ tumor bioaccumulation period and lowered systemic absorption, thus improving the situation in vivo. Moreover, a recent study demonstrated that the antitumor activity, referring to the survival benefit and the rate of complete responses by combined local radiotherapy with the HPV16 E6/E7 RNA-LPX vaccine, was synergized in mouse models of HPV16+ cancer [55]. The E7 RNA-LPX vaccine leads to intratumoral E7-specific CD8+ T-cell responses, and local radiotherapy can further enhance these effects.

Photodynamic and photothermal therapy (PDT/PTT) are techniques to eliminate cancer cells by generating reactive oxygen species (ROS) or by converting photon energy into thermal energy. To destroy cells, cytotoxic ROS are generated by the reaction between the photosensitizer and the oxygen in the surrounding tissue under light. Moreover, PDT treatment can also trigger antitumor immunity [1]. This has been shown to be a promising and noninvasive approach for several cancer treatments [126]. In recent years, nanomaterials have gained popularity for their applications in PDT/PTT due to their numerous advantages, such as repeatable synthesis, high affinity, and adaptability to various modification techniques. For example, chlorin e6 (Ce6) is a highly effective photosensitizer that can be employed in several studies for optical imaging and PDT/PTT. Song et al. developed nanotechnology-based drug delivery systems (CECMa NPs) based on cisplatin (CDDP) and metformin (chemotherapeutic sensitizer), of which Ce6 and polyethylene glycol diamine (PEG) were synthesized as the shell, and an anti-LDLR antibody was modified on the surface. In in vivo studies, CECMa NPs increased the phototherapy effects on HNSCC and reduced the systemic toxicity of chemotherapy [127]. In addition, given the important role of ICB treatment, recent efforts have been made to combine ICB treatment with PDT/PTT, which can enhance the antitumor immune response, as reviewed elsewhere [128]. Moreover, photoimmunotherapy (PIT) that combines phototherapy and immunotherapy has been developed with the aim of eliminating both the primary tumor and recurrence or metastasis of cancer (Figure 2B). RM-1929 PIT, which uses cetuximab sarotalocan sodium, an antibody targeting EGFR conjugated with a light-activatable dye, combined with a laser system to target only tumor cells, is emerging as an important treatment strategy for recurrent HNSCC. In a recent phase 1/2a trial in patients with locoregional recurrent HNSCC, RM-1929 PIT demonstrated a safety profile [129]. A novel proof-of-concept photodynamic image-guided surgery was also developed utilizing EGFR monoclonal antibody conjugated with the fluorophore IRDye800CW in an HNSCC mouse model [130]. To integrate a novel immunotherapy with phototherapy, nanotechnology has been employed due to its multifunctional ability, such as drug-loading capacity, site-specific delivery, and ability to serve as photothermal agents or photosensitizers [131]. For example, a nanoplatform was developed based on mesoporous copper sulfide nanoparticles (CuS) conjugated with the tumor target ligands folic acid (FA) and docetaxel and further fabricated with polyethylenimine-protoporphyrin IX (PEI-PpIX) conjugates to improve water solubility for successful CpG delivery [132]. These nanocomposites demonstrate a good PPT and PDT effect in a 4T1 tumor model. Moreover, when anti-PD-L1 antibody (aPD-L1) is combined with these nanocomposites, aPD-L1 + PDT + PTT may increase infiltration of CTLs and suppress myeloid-derived suppressor cells (MDSCs) as well as polarize MDSCs toward the M1 phenotype in tumor sites [132]. Although current PDT/PTT/PIT therapies are not designed, especially for HPV-related HNSCC, research on their application to HPV-related cancer is clinically meaningful and warrants investigation.

**Table 2:** Nanovaccine preclinical studies for HPV-related HNSCC.

Antigen	Type	Combination	Adjuvant	Delivery Platform	Cancer Model	References
HPV-16 E7	Peptide	Anti-41BB	CpG-B 1826 oligonucleotide	Poly (propylene sulfide) nanoparticle	TC-1 tumor-bearing mice	[133]
HPV-16 E7	Peptide			R-DOTAP cationic lipid nanoparticle	TC-1 tumor-bearing mice	[56]
HPV-16 E7	mRNA	PD-L1 antibody		DOTMA/DOPE liposome	TC-1 & C3 tumor-bearing mice	[41]
HPV-16 E7	DCs		Poly (I:C)	PLGA nanoparticles	TC-1 tumor-bearing mice	[134]
HPV-16 E6/E7	Protein	Anti PD-L1, Cisplatin	Poly (I:C), R848, CpG ODNs	PLGA nanoparticles	TC-1 tumor-bearing mice, cynomolgus monkey	[44]
HPV-16 E6/E7	RNA			DOTMA/DOPE liposome	TC-1 tumor-bearing mice	[54]
HPV-16 E7	Peptide		MPLA, CpG	Synthetic high-density lipoprotein nanodisc	TC-1 tumor-bearing mice	[57]
HPV-16 E7	Peptide		Polyethyleneimine, GM-CSF, CPG-ODN	Mesoporous silica micro-rod	TC-1 tumor-bearing mice	[60]
HPV-16 E7	Peptide			Q11 peptide assembled nanofiber	TC-1 tumor-bearing mice	[64]
HPV-16 E7	Peptide		Poly (I: C), CpG-ODN	Hyaluronic acid-modified cationic lipid-PLGA hybrid nanoparticles	TC-1 tumor-bearing mice	[135]
HPV-16 E7	RNA			Heterocyclic lipid nanoparticle	TC-1 tumor-bearing mice	[42]
HPV-16 E7	DNA/Protein			Supercharged green fluorescent protein	TC-1 tumor-bearing mice	[136]
HPV-16 E7	Protein	Anti-CD40	Pam3CSK4, Poly (I: C)	PLGA nanoparticle	TC-1 tumor-bearing mice	[137]
HPV-16 E7	Peptide		GM-CSF	HIV tat peptide	TC-1 tumor-bearing mice	[65]
HPV-16 E7	Peptide		MPLA	PEG-PE micelle	TC-1 tumor-bearing mice	[138]
HPV-16 E7	Protein			Bacterial outer membrane vesicles	TC-1 tumor-bearing mice	[43]
HPV-16 E6/E7	Peptide	Anti-PD-L1		Nanosatellite	Mouse HNSCC (PCI-13, UMSCC22b, UMSCC47, and FaDu cells)	[63]
HPV-16 E7	DNA			Branched amphiphilic peptide capsules	TC-1 tumor-bearing mice	[139]
HPV-16 E7	Peptide			Virus-like particles	TC-1 tumor-bearing mice	[66]
HPV-16 E7	Peptide			Liposome	TC-1 tumor-bearing mice	[140]
HPV-16 E7	Peptide		GM-CSF, CpG-ODN	Mesoporous silica rods	Mouse HNSCC (MOC2-E6E7 cells)	[61]
HPV-16 E7	Peptide		R837	Crosslinked BSA- E7	TC-1 tumor-bearing mice	[68]
HPV-16 E7	mRNA	Local Radiotherapy		DOTMA/DOPE liposome	TC-1 tumor-bearing mice	[55]
HPV-16 E6/E7	Peptide		PHAD-3D6A, QS-21	CPQ liposome	TC-1 tumor-bearing mice	[141]
HPV-16 E7	Peptide	Surgery	CpG-B 1826 oligonucleotide	Poly (propylene sulfide) nanoparticle	mEERL95 tumor-bearing mice	[69]
HPV-16 E7	Peptide			manganese-doped silica nanoparticles	TC-1 tumor-bearing mice	[62]
HPV-16 E7	Peptide		CpG-ODN	Mannose-Modified Liposome	TC-1 tumor-bearing mice	[142]
HPV-16 E6/E7	Peptide	Bintrafusp alfa (M7824), NHS- IL12		R-DOTAP containing lipid nanoparticle	TC-1 and mEER tumor-bearing mice	[47]
HPV -16 L1/E6/E7	DNA			Archaeosome	TC-1 tumor-bearing mice	[143]
HPV -16 E7	Peptide	Anti-PD-1 (G4C2)	CpG-1826	Spycatcher modified Ferritin nanoparticle	TC-1 tumor-bearing mice	[144]

## 8. Summary

This review highlights strategies such as vaccines or combination therapy for developing nanotherapeutics for patients affected by HR-HPV-driven HNSCC. The various benefits and characteristics of these nanocarriers summarized in this review that form the knowledge basis are discussed. The major advantages of nanotherapeutics include the multifunctionality of drug-loading capacity, targeted delivery and prolonged circulation time, synergistic effects with pharmacological and physical cotreatments, and, therefore, improved drug delivery, reduced toxicity, and enhanced therapeutic efficacy. Nanocarriers can also sensitize cancer cells to conventional therapeutic agents by modulating the TME and mechanism-based specific targets, i.e., by targeting CSCs specifically. Additionally, utilizing nanoparticles for manufacturing the spatiotemporal delivery of CRISPR nanocarriers or mRNA and siRNA technologies is promising for developing combination therapy or cancer vaccines. In conclusion, nanomedicine-based approaches are strongly encouraged for clinical oncology and have the potential to develop treatments for HR-HPV-driven HNSCC.

## Figures and Tables

**Table 1:** Nanovaccine clinical trials studies for HPV-related HNSCC.

Vaccine	Type	Combination	Phase	Antigen	Disease	Delivery Platform	Status (as of 1 September 2022)	Identifier
PDS0101	Peptide	M7824 (Targeting both PD-L1 and TGFβ), NHS-IL12 (immunocytokine)	I/II	HPV16 E6/E7	locally advanced or metastatic HPV associated cancer	R-DOTAP containing lipid nanoparticle	Recruiting	NCT04287868
PDS0101	Peptide	Pembrolizumab	II	HPV16 E6/E7	HPV16+ Recurrent and/or Metastatic HNSCC	R-DOTAP containing lipid nanoparticle	Recruiting	NCT04260126
PDS0101	Peptide	Pembrolizumab	I/II	HPV16 E6/E7	Locally Advanced HPV Associated Oropharynx Cancer	R-DOTAP containing lipid nanoparticle	Recruiting	NCT05232851
BNT113	mRNA	Anti-CD40	I/II	HPV 16 E6/E7	Advanced HPV16+ cancer	RNA-lipoplex	Recruiting	NCT03418480
BNT113	mRNA	Pembrolizumab	II	HPV 16 E6/E7	Unresectable recurrent or metastatic HPV16+ and PD-L1+ HNSCC	RNA-lipoplex	Recruiting	NCT04534205

## Supplementary Materials

The following supporting information can be downloaded at: https://www.mdpi.com/article/10.3390/v14122824/s1, Supplemental Table S1. Clinical trials of vaccines or vaccines included combination therapies for HPV-related HNSCC. References [145,146,147,148,149] are cited in Supplementary Materials.
