# Supplementary material for: Advanced Nanomedicine for High-Risk HPV-Driven Head and Neck Cancer
Source: Viruses. 2022 Dec 19;14(12):2824. doi: 10.3390/v14122824 (PMC9788019; doi:10.3390/v14122824)
Supplement: Supplementary file 1 [file viruses-14-02824-s001.zip › viruses-2031707-supplementary.pdf]

Supplemental Table S1 Clinical trials of vaccines or vaccines included combination therapies for HPV-related HNSCC.

Type	Identifier	Vaccine	Combination	Phase	Antigen	Disease	Delivery Platform	Status (as of 1 September 2022)
peptide								
	NCT02865135	DPX-E7		Ib/II	HPV16 E7	Incurable HPV16+ head and neck, cervical or anal cancer (positive for HLA-A*02)		Active, not recruiting
	NCT04398524	ISA101b	Cemiplimab (anti-PD1)	II	HPV16 E6/E7	Recurrent/Metastatic HPV16+ Squamous Cell Carcinoma of the Oropharynx		Recruiting
	NCT03669718	ISA101b	Cemiplimab (anti-PD1)	II	HPV16 E6/E7	Recurrent/Metastatic HPV16+ Squamous Cell Carcinoma of the Oropharynx		Recruiting
	NCT03258008	ISA101b	Utomilumab (4-1BB/CD137 agonist)	II	HPV16 E6/E7	HPV-16+ incurable Oropharyngeal Cancer		Completed
	NCT04369937	ISA101b	IMRT(Intensity Modulated Radiotherapy)， Pembrolizumab (anti-PD1)， Cisplatin	II	HPV16 E6/E7	Intermediate Risk HPV-16 Associated HNSCC		Recruiting
	NCT02426892[45,48]	ISA101	Nivolumab (anti-PD1)	II	HPV16 E6/E7	HPV-16+ Incurable Solid Tumors		Completed
	NCT03821272	PepCan		I/II	HPV16 E6	Head and neck cancer patients in remission regardless of HPV status		Recruiting
	NCT04287868	PDS0101	M7824 (a Bifunctional Fusion Protein Targeting both PD-L1 and TGFβ), NHS-IL12 (immunocytokine)	I/II	HPV16 E6/E7	locally advanced or metastatic HPV associated cancer	R-DOTAP containing lipid nanoparticle	Recruiting
	NCT04260126	PDS0101	Pembrolizumab (anti-PD1)	II	HPV16 E6/E7	HPV16 + Recurrent and/or Metastatic HNSCC	R-DOTAP containing lipid nanoparticle	Recruiting
	NCT05232851	PDS0101	Pembrolizumab (anti-PD1)	I/II	HPV16 E6/E7	Locally Advanced Human Papillomavirus-Associated Oropharynx Cancer	R-DOTAP containing lipid nanoparticle	Recruiting

	NCT02821494	Hespecta	Amplivant® (synthetic TLR2 ligand)	I	HPV16 E6	HPV16+ Tumors or Premalignant Lesions		Completed
	NCT00257738[145]	GL-0810 GL-0817		I	HPV16 MAGE-A3	Recurrent, progressive or metastatic HNSCC, positive for either MAGE-A3 or HPV16		Completed
	NCT02526316	P16_37-63	cisplatin	I	p16INK4a	HPV- and p16INK4a-positive cervical, vulvar, vaginal, penile, anal or head and neck cancer		Completed
	NCT01462838[146]	P16_37-63		I	p16INK4a	HPV- and p16INK4a-positive cervical, vulvar, vaginal, penile, anal or head and neck cancer		Completed
	NCT03946358	UCPVAX	Atezolizumab (anti-PD-L1)	II	telomerase	HPV16+ cancers (Anal cancer, Head and Neck carcinoma, cervical and vulvar carcinoma)		Recruiting
RNA								
	NCT03418480	BNT113	Anti-CD40	I/II	HPV 16 E6/E7	Advanced HPV16+ cancer (HNSCC, anogenital, penile, cervical)	RNA-lipoplex	Recruiting
	NCT04534205	BNT113	Pembrolizumab (anti-PD1)	II	HPV 16 E6/E7	Unresectable recurrent or metastatic HPV16+ and PD-L1+ HNSCC	RNA-lipoplex	Recruiting
DNA								
	NCT02163057[51]	INO-3112 (VGX-3100 + INO-9012)	Cohort 1: before and after surgery. Cohort 2: after chemoradiation therapy	I/II	HPV 16 and HPV18 E6/E7	HPV+ HNSCC		Completed
	NCT03162224	INO-3112	Durvalumab （anti -PD-L1）	Ib/IIa	HPV 16 and HPV18 E6/E7	Recurrent/Metastatic HPV Associated Head and Neck Squamous Cancer		Completed
	NCT01493154	pNGVL-4a-CRT/E7 (detox) DNA Vaccine	Cyclophosphamide	I	HPV16 E7	HPV-16 Associated Head and Neck Cancer		Terminated

	NCT05286060	GX-188E	Pembrolizumab (anti-PD1) GX-I7	II	HPV16 and HPV18 E6/E7	Advanced, Non-Resectable HPV 16 and/or 18 + Head and Neck Cancer	recruiting
	ACTRN12618000140257[147]	AMV002		I	HPV16 E6/E7	HPV-associated oropharyngeal squamous cell carcinoma	completed
	ACTRN12620000406909	AMV002	Durvalumab （anti -PD-L1）	Ib	HPV16 E6/E7	recurrent and/or metastatic HPV-related, p16 positive oropharyngeal squamous cell carcinoma	completed
	NCT05280457	GX-188E	Nivolumab (anti-PD1) GX-I7	II	HPV16 and HPV18 E6/E7	Advanced, Resectable HPV Type 16 and/or 18 Positive Head and Neck Cancer	Recruiting
Viral vector							
	NCT05108870	HB-201, HB-202	Carboplatin， Paclitaxel， Transoral Robotic Surgery	I/II	HPV16 E6/E7	Locoregional HPV16+ Oropharyngeal Cancer	Recruiting
	NCT04630353	HB-201		I	HPV16 E6/E7	Newly diagnosed HPV16+ Oropharynx or locally advanced cervical cancer	Recruiting
	NCT04180215	HB-201, HB-202	Immune checkpoint inhibitor regimen per standard of care	I/II	HPV16 E6/E7	HPV16+ Recurrent/Metastatic HNSCC and other cancers.	Recruiting
	NCT04432597	PRGN-2009	M7824 (a Bifunctional Fusion Protein Targeting both PD-L1 and TGFβ)	I/II	HPV16 and HPV18 E6/E7	locally advanced or metastatic HPV associated cancer, stage II or III p16-positive oropharyngeal cancer	Recruiting
	NCT03260023	TG4001	Avelumab (anti-PD-L1)	I/II	HPV16 E6/E7	HPV16 + R/M Cancers	Recruiting
Bacterial vector							
	NCT02002182	ADXS11-001		II	HPV16 E7	stage I-IV OPSCC prior to ablative transoral robotic surgery.	Completed
	NCT01598792	ADXS11-001		I	HPV16 E7	HPV-16+ Oropharyngeal Carcinoma	Terminated
	NCT02291055	ADXS11-001	MEDI4736	I/II	HPV16 E7	Previously Treated Locally Advanced or Metastatic Cervical or HPV+ HNSCC	Unknown
Cell							

	NCT00019110	HPV E6 or E7 peptide pulsed peripheral blood mononuclear cells		I	HPV16 E6/E7	advanced or recurrent cancer of the cervix, vagina, penis, anus, esophagus, or head and neck.	Completed
	NCT02858310[148]	E7 TCR cells	Aldesleukin, Fludarabine, Cyclophosphamide	I/II	HPV16 E7	Metastatic or refractory/recurrent HPV-16+ cancers	Recruiting
	NCT00404339[149]	mutant p53 peptide pulsed dendritic cell vaccine	tetanus toxoid helper peptide	I	p53	squamous cell carcinoma of the head and neck.	Completed
	NCT05357898	SQZ-eAPC-HPV	Pembrolizumab	I/II	HPV16 E6 /E7, CD86 costimulatory factor, membrane-bound IL-2 and IL-12 cytokines	HPV16+ Recurrent, Locally Advanced or Metastatic Solid Tumors	Recruiting
	NCT04084951	SQZ-PBMC-HPV	Atezolizumab(anti PD-L1, Ipilimumab(anti-CTLA-4)， Nivolumab(anti-PD1)	I	immunogenic epitopes of HPV16	HLA-A*02+ Patients With HPV16+ Recurrent, Locally Advanced or Metastatic Solid Tumors	Recruiting
	NCT04892043	SQZ-AAC-HPV	Ipilimumab(anti-CTLA-4)， Nivolumab(anti-PD1)	I	immunogenic epitopes of HPV16	HLA-A*02+ Patients With HPV16+ Recurrent, Locally Advanced or Metastatic Solid Tumors	Recruiting
